# Hyperthermia Differently Affects Connexin43 Expression and Gap Junction Permeability in Skeletal Myoblasts and HeLa Cells

**DOI:** 10.1155/2014/748290

**Published:** 2014-07-20

**Authors:** Ieva Antanavičiūtė, Vida Mildažienė, Edgaras Stankevičius, Thomas Herdegen, Vytenis Arvydas Skeberdis

**Affiliations:** ^1^Institute of Cardiology, Lithuanian University of Health Sciences, Sukilėli*ų* Avenue 17, 50009 Kaunas, Lithuania; ^2^Faculty of Natural Sciences, Vytautas Magnus University, 44404 Kaunas, Lithuania; ^3^Institute of Physiology and Pharmacology, Lithuanian University of Health Sciences, 44307 Kaunas, Lithuania; ^4^Institute of Experimental and Clinical Pharmacology, Kiel University, Hospitalstraße 4, 24105 Kiel, Germany

## Abstract

Stress kinases can be activated by hyperthermia and modify the expression level and properties of membranous and intercellular channels. We examined the role of c-Jun NH_2_-terminal kinase (JNK) in hyperthermia-induced changes of connexin43 (Cx43) expression and permeability of Cx43 gap junctions (GJs) in the rabbit skeletal myoblasts (SkMs) and Cx43-EGFP transfected HeLa cells. Hyperthermia (42°C for 6 h) enhanced the activity of JNK and its target, the transcription factor c-Jun, in both SkMs and HeLa cells. In SkMs, hyperthermia caused a 3.2-fold increase in the total Cx43 protein level and enhanced the efficacy of GJ intercellular communication (GJIC). In striking contrast, hyperthermia reduced the total amount of Cx43 protein, the number of Cx43 channels in GJ plaques, the density of hemichannels in the cell membranes, and the efficiency of GJIC in HeLa cells. Both in SkMs and HeLa cells, these changes could be prevented by XG-102, a JNK inhibitor. In HeLa cells, the changes in Cx43 expression and GJIC under hyperthermic conditions were accompanied by JNK-dependent disorganization of actin cytoskeleton stress fibers while in SkMs, the actin cytoskeleton remained intact. These findings provide an attractive model to identify the regulatory players within signalosomes, which determine the cell-dependent outcomes of hyperthermia.

## 1. Introduction

Skeletal myoblasts (SkMs) have been investigated during the last decade for their potential in several fields of regenerative medicine. SkMs have been applied for the treatment of myocardial infarction (MI), Duchenne's muscular dystrophy, Chagas' disease, muscle trauma, and so forth [1–5]. Thus, SkMs are considered to be appropriate candidates for stem cell therapy due to their high proliferative potential, resistance to ischemia, simple isolation from muscular biopsy, and absence of tumorigenicity as well as of immunological and ethical concerns. Animal studies have shown positive effects of autologous SkM transplantation on the cardiac function [1, 6–8], but controversial data were obtained from phase I clinical trials, which failed to demonstrate the functionally effective postinfarctional heart regeneration with SkMs [9]. A number of issues need to be resolved concerning stem cell transplantation. Implanted cells show a low rate of incorporation and viability in the ischemic environment. For instance, Suzuki et al. have shown that only 7.4% of SkMs survived in mice hearts 72 h after injection [10]. MI is accompanied by adverse side effects such as inflammation, hypoxia, and impaired metabolism [11–13]. The main disadvantage of SkM application is the increased risk of ventricular tachyarrhythmias [14]. For the proper excitation of the heart, engrafted cells need to establish functional intercellular communication with host cardiomyocytes [15]. Gap junction (GJ) channels are composed of two opposing hemichannels in contiguous cells and provide a direct pathway for electrical and metabolic intercellular communication [16]. Six connexin (Cx) subunits oligomerize into connexon, which is called a hemichannel after insertion into plasma membrane. The family of connexin genes consists of 21 genes in the human genome. Cx43 is the most abundant connexin protein in the ventricular myocardium, responsible for gap junction intercellular communication (GJIC) between working myocytes [17, 18]. Nondifferentiated SkMs also express endogenous Cx43 that is important not only in coupling with cardiac myocytes but also in the differentiation of SkMs and the regeneration of skeletal muscle [19]. Unfortunately, Cx43 is downregulated during SkM differentiation [20, 21]. Induced expression of Cx43 in SkMs may serve as an appropriate strategy to improve their therapeutic benefit. At least, genetically modified myoblasts expressing Cx43 have been shown to decrease the arrhythmogenicity [22–24]. Many other factors, such as antiapoptotic or angiogenesis-initiating genes [25, 26], preconditioning with growth factors [15, 27–29], or heat treatment [30–33], have been shown to contribute to the improvement of the efficiency of stem cell therapy. Inflammation and cell survival during cardiac ischemia/reperfusion (I/R) injury is essentially regulated by mitogen activated protein kinase (MAPK) signaling pathways. Three MAPK subfamilies are known to play a major role in the I/R heart: extracellular signal-regulated kinases 1/2 (ERK1/2), c-Jun NH_2_-terminal kinase (JNK), and p38 MAPK [34]. The exposure of rat cardiomyocytes to ischemia activates ERK, p38, and JNK [35]. The activation of ERK protects cardiomyocytes from apoptosis and reduces infarct size [36, 37], but the data on the impact of p38 and JNK activation on the cardiac function during I/R are conflicting. On the one hand, the activation of p38 and JNK induces apoptosis of cardiomyocytes and exacerbates heart injury after I/R [35, 38–40], but on the other hand, there is evidence of their protective mechanisms [41–43]. Moreover, the activation of ERK or the inhibition of JNK and p38 pathways has been reported to improve the heart function after MI (or I/R) [34, 44–46].

JNK can be activated by inflammatory cytokines and numerous stressors such as heat shock, oxidative stress, or DNA damage, which follow I/R [47–50]. I/R and surgical interventions evoke inflammatory responses that activate JNK and/or other kinases. Consequently, the expression of Cxs and the properties of membranous and intercellular channels can be modified by stress kinases. Indeed, the activation of JNK up- or downregulates the expression of Cx43 depending on the cell type [51, 52].

Here we found that hyperthermia induced JNK-dependent changes in Cx43 expression and GJIC in HeLa cells expressing exogenous Cx43-EGFP and in SkMs expressing endogenous Cx43.

In parallel, hyperthermia caused JNK-dependent disorganization of the F-actin network in HeLa Cx43-EGFP cells, while in SkMs it remained unaltered.

## 2. Methods

The investigation conforms to the European Community guidelines for the care and use of animals (86/609/CEE, CE Off J no. L358, December 18, 1986). The license for the use of laboratory animals (no. 0171, October 31, 2007) was received from the Lithuanian Food and Veterinary Service.

### 2.1. Isolation and Culturing of Skeletal Myoblasts

New Zealand white rabbits of both genders, aged 6 to 12 months and weighing 3.0 to 3.5 kg, were used in this study. A piece of femoral skeletal muscle (0.5 cm^3^) was taken under general anesthesia and placed into a transportation medium (Iscove's modified Dulbecco's medium (IMDM) with antibiotics, penicillin 300 U/mL, and streptomycin 300 *μ*g/mL). The tissue was mechanically cut into small pieces and treated with an enzyme mixture: 1 mg/mL collagenase (type V), 0.3 mg/mL hyaluronidase (type IV-S), 0.125% trypsin, and 0.1% EDTA in PBS. After washing with IMDM, cells were seeded into flasks with a growing medium (IMDM with 10% FBS, penicillin 100 U/mL, and streptomycin 100 *μ*g/mL). All chemicals were purchased from Sigma Aldrich Corp. (Steinheim, Germany).

### 2.2. HeLa Cx43-EGFP Growing Conditions

Cx43-EGFP-expressing HeLa cells were kindly donated by Professor Feliksas Bukauskas (Yeshiva University, New York, NY, USA). HeLa Cx43-EGFP cells were grown in a Dulbecco's Modified Eagle Medium (DMEM) supplemented with 10% FBS and antibiotics (penicillin 100 U/mL and streptomycin 100 *μ*g/mL).

### 2.3. Immunocytochemistry

Cells were grown on glass coverslips 1 cm in diameter and fixed with 4% paraformaldehyde for 15 min. Then cells were washed with PBS and incubated in PBS with 0.2% Triton X-100 for 3 min to permeabilize membranes. Cells were incubated with primary antibodies against desmin, myogenin (Abcam, Cambridge, UK), Cx43 (Transduction Laboratories, Lexington, KY, USA), or Cx45 (Invitrogen, CA, USA) for 1 h or with Alexa Fluor 594 phalloidin (Invitrogen, CA, USA) for 30 min at 37°C. Then cells were rinsed in PBS with 1% BSA and incubated with secondary goat anti-mouse IgG H&L antibodies conjugated with Cy5 or with donkey anti-rabbit IgG H&L Alexa Fluor 488 (Abcam, Cambridge, UK). Coverslips were attached with a Vectashield Mounting Medium with DAPI (Vector Laboratories, CA, USA). The analysis was performed with an inverted fluorescence microscope Olympus IX81 (Olympus Europa holding Gmbh, Hamburg, Germany) equipped with an Orca-R^2^ cooled digital camera (Hamamatsu Photonics K.K., Japan), the fluorescence excitation system MT10 (Olympus Life Science Europa Gmbh, Hamburg, Germany), and the fluorescence imaging system XCELLENCE (Olympus Soft Imaging Solutions Gmbh, München, Germany).

### 2.4. Immunoblot Assay

Cells were lysed in ice-cold lysis buffer (50 mM Tris pH 7.5, 50 mM NaCl, 1 mM EDTA, 1 mM EGTA, 50 mM NaF, 1% Triton X-100, 1 mM *β*-glycerophosphate, 1 mM Na_3_VO_4_, and protease inhibitor cocktail 1 : 50). Equal amounts of protein were separated by SDS-PAGE on 10% acrylamide gels. Protein concentrations were estimated by the Bradford assay (Bio-Rad, CA, USA). Proteins separated in gel were transferred to the PVDF membrane (GE Healthcare, CA, USA) and then were blocked with blocking buffer containing 5% low fat milk or 5% BSA in TBST (50 mM Tris pH 7.6, 150 mM NaCl, 0.05% Tween-20) for 1 h at room temperature. Membranes were probed with primary antibodies against Cx43 (Transduction Laboratories, Lexington, KY, USA), GFP (Invitrogen, CA, USA), p-JNK, p-c-Jun, and AKT *β* (Cell Signalling Technology, MA, USA) in 5% low fat milk or 5% BSA in TBST for 24 h at 4°C and after washing with TBST solution were incubated with secondary antibodies, goat anti-rabbit Fab fragment of IgG, and goat anti-mouse conjugated with alkaline phosphatase (Invitrogen, CA, USA), for 1 h at room temperature. The proteins were visualized by the alkaline phosphatase method (Carl Roth GmbH & Co., KG Karlsruhe, Germany).

### 2.5. Hyperthermic Conditions and Inhibition of JNK Activity

Two hours before the beginning of experiments, cells were washed with freshly equilibrated growth media (37°C, 5% CO_2_). Subconfluent cultured cells (SkMs and HeLa cells) were subjected to hyperthermia (42°C for 2, 6 or 24 h) in the 5% CO_2_ incubator. XG-102, a selective JNK inhibitor [53, 54], was applied 30 min before the induction of hyperthermia treatment (4 *μ*M, 37°C, 5% CO_2_). For the respective control measurements, cells were kept at 37°C for the same periods as those under hyperthermic conditions.

### 2.6.  Measurement of GJIC

For fluorescence recording, cells grown onto glass coverslips were transferred to an experimental chamber with constant flow-through perfusion mounted on the stage of an inverted microscope Olympus IX81. Appropriate excitation and emission filters (Chroma technology, Brattleboro, VT) were used to image Lucifer Yellow CH dilithium salt dye (LY; MW = 457.25; net charge −2, *λ*_ex_ = 436 nm, *λ*_em_ = 480 nm). For dye transfer studies, LY was introduced into the first cell of a contiguous cell row through a patch pipette in the whole-cell voltage-clamp mode. Typically, this resulted in rapid loading of the first cell, followed by dye transfer via GJs to the neighboring cells. Fluorescence kinetics was evaluated using the XCELLENCE software (background subtracted). Microelectrode resistance was 2-3 MΩ. Internal solution: NaCl 130 mM, KCl 30 mM, NaAsp 10 mM, CaCl_2_ 0.26 mM, Hepes 5 mM, EGTA 2 mM, TEA 5 mM, MgCl_2_ 1 mM, MgATP 3 mM (pH 7.3), and 2 mM LY; external solution: NaCl 140 mM, KCl 4 mM, CaCl_2_ 2 mM, MgCl_2_ 1 mM, Hepes 5 mM, glucose 5 mM, and pyruvate 2 mM (pH 7.4). LY diffusion was registered for 30 min. To minimize dye bleaching, images were taken every 2 min at low excitation intensity and 200-ms exposure.

### 2.7. Scrape-Loaded Dye Transfer Measurements

GJIC was studied by measuring scrape-loaded LY transfer. Grown to confluence on the glass coverslips, cells were washed with Ca^2+^-free PBS. LY solution (100 *μ*L, 2 mM) was applied on each coverslip, and several cuts were made on the monolayer with a surgical blade to introduce the dye into the damaged cells. After incubation for 3 min at room temperature, cells were rinsed three times with PBS containing 2 mM Ca^2+^, before fixing with 4% paraformaldehyde in PBS for 15 min [55]. LY transfer between cells was evaluated by measuring the exponential decay of LY fluorescence (FD_50_) perpendicularly from the scrape line. Fluorescence imaging and measurements were performed using the same hardware and software as described in Section 2.3.

### 2.8. Statistical Analysis

Data are expressed as mean of at least 3 independent experiments ± standard error. The unpaired Student's *t*-test was used for western blot (WB) quantitative evaluation. For comparison of GJIC data, the density of hemichannels in cell membranes, and the number of channels in junctional plaques (JPs), we used ANOVA analysis with the post hoc Dunnett's or Bonferroni's test. Differences were considered statistically significant at *P* < 0.05.

## 3. Results

### 3.1. Characterization of Skeletal Myoblasts

**Figure 1:**
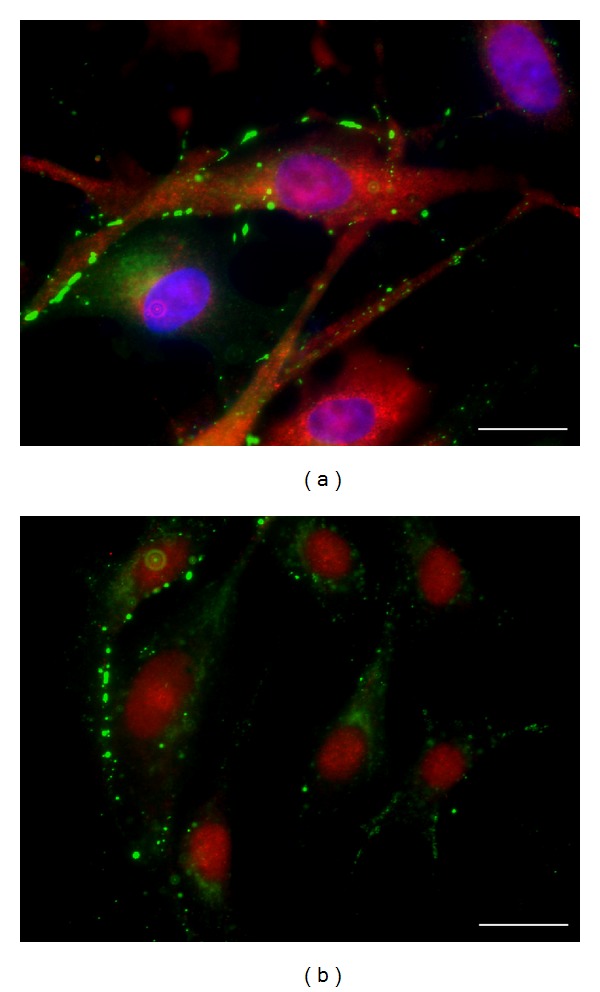
Characterization of rabbit SkMs. (a) Immunostaining of desmin (red) and endogenous Cx43 (green). Nuclei are labeled with DAPI (blue). (b) Immunostaining of myogenin (red) and endogenous Cx43 (green). Scale bar 20 *μ*m.

Desmin, a muscle-specific marker, demonstrated the purity of the experimental cultures of SkMs obtained from the rabbit femoral muscle [56]. Almost all cells (99%) were desmin positive (Figure 1(a)). Myogenin, a marker of myogenic differentiation, was present in around 80% of the cell nuclei (Figure 1(b)). The majority of the SkMs expressed endogenous Cx43 (Figures 1(a) and 1(b)). Araya et al. have suggested that, in addition to Cx43, the skeletal muscle and primary cultures of myoblasts of the mice may express Cx45 [19]; however, in our SkMs, Cx45 expression was not detected by immunofluorescence experiments (data not shown).

### 3.2. Hyperthermia and SkM Intercellular Permeability

**Figure 2:**
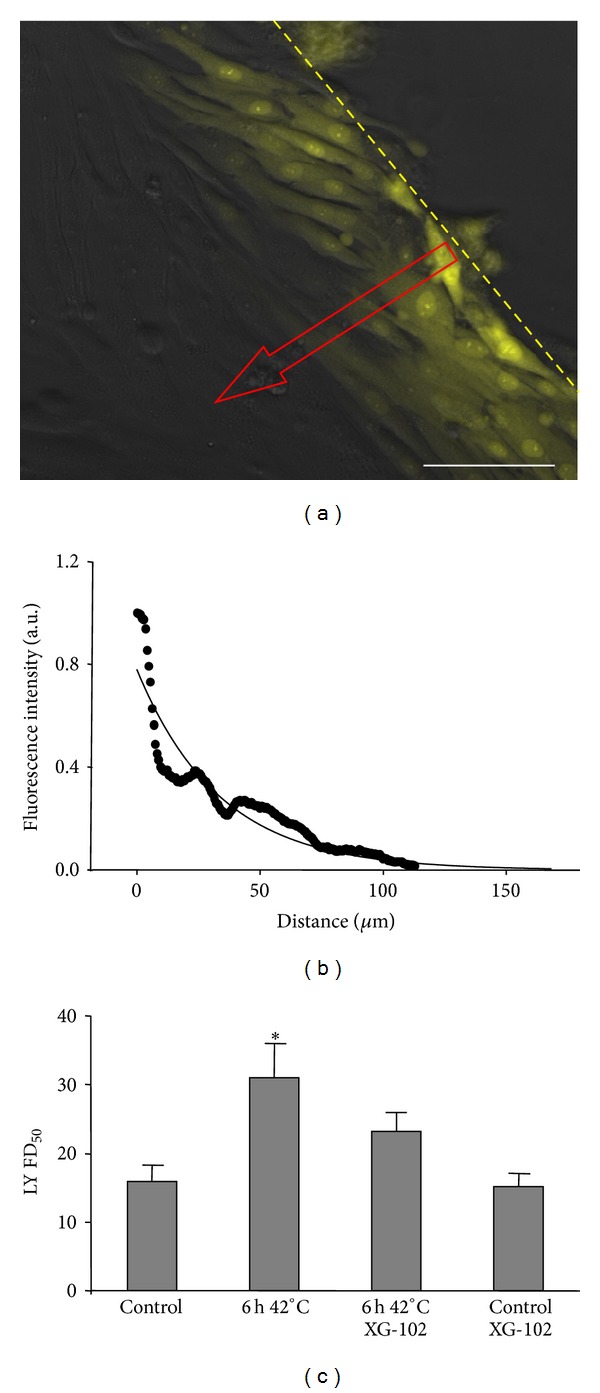
Effect of hyperthermia on Cx43 GJIC in SkMs. (a) LY diffusion between SkMs was measured by the scrape-loaded dye transfer technique (dashed line indicates the scrape margin; arrow shows the measurement direction of the LY diffusion). (b) Kinetics of scrape-loaded LY diffusion under control conditions, measured 3 min after LY application. Raw data were fitted with an exponential decay function. (c) Comparison of scrape-loaded LY diffusion under control and hyperthermia conditions in the presence and absence of XG-102 (4 *μ*M). FD_50_ was 15.7 ± 2.4, 30.3 ± 5.4, 15.1 ± 1.6, and 22.5 ± 2.8 *μ*m, respectively. *n* = 10 in each set of experiments;  * = *P* < 0.05, ANOVA followed by post hoc Dunnet's test. Scale bar 100 *μ*m.

Since undifferentiated SkMs express the endogenous Cx43 protein, we used a scrape-loaded dye transfer technique to evaluate GJIC. FD_50_ of LY was measured perpendicularly from the scrape line (Figures 2(a) and 2(b)). The distance of LY diffusion was 2 times longer in hyperthermically incubated cells compared with control cells. FD_50_ was 15.7 ± 2.4 *μ*m and 30.3 ± 5.4 *μ*m in control and after 6 h at 42°C, respectively (*P* < 0.05). Treatment with XG-102 (4 *μ*M), a JNK-inhibitor, had no effect on the FD_50_ under control conditions but prevented the hyperthermia-induced increase in FD_50_ (Figure 2(c)).

### 3.3. Hyperthermia and Total Cx43 Protein Expression in SkMs

**Figure 3:**
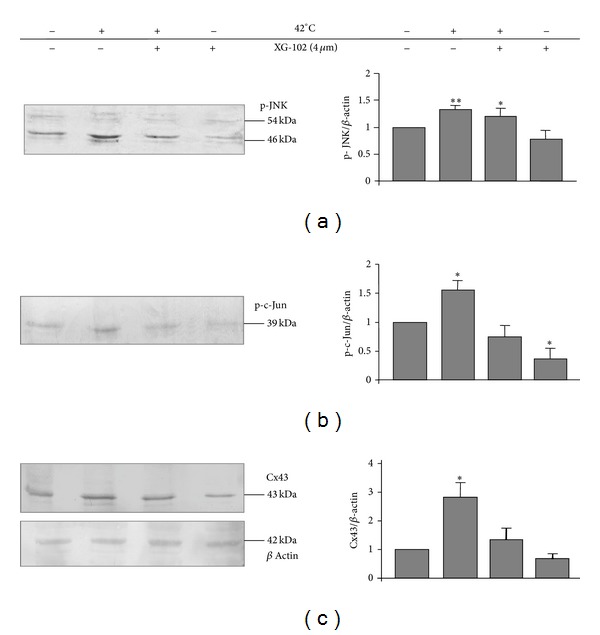
Effect of hyperthermia on the total expression of p-JNK (a), p-c-Jun (b), and endogenous Cx43 protein (c) in SkMs. *n* = 3 in each set of experiments;  * = *P* < 0.05;  ** = *P* < 0.01, Student's *t*-test.

Thus, hyperthermia treatment caused the JNK-dependent amelioration of GJIC in the SkM culture. To determine whether these changes were caused by an increase in total Cx43 protein expression, the total amounts of activated JNK (p-JNK), activated c-Jun (p-c-Jun), and Cx43 protein were measured by WB under all experimental conditions described above. Hyperthermia activated JNK signaling in SkMs resulting in significantly increased levels of phosphorylated JNK, phosphorylated c-Jun, and Cx43 protein by 1.3-, 1.6-, and 2.8-fold, respectively (Figure 3). XG-102 did not affect JNK phosphorylation since the phosphorylation domain differs from the catalytic domain. In contrast, XG-102 significantly inhibited the basal and hyperthermia-stimulated activity of the JNK substrate c-Jun (Figure 3(b)). In addition, XG-102 prevented the upregulation of Cx43 by hyperthermia (Figure 3(c)).

### 3.4. The Effect of Hyperthermia and JNK on Cx43-EGFP GJ Plaque Size and Membranous Hemichannel Density in HeLa Cells

HeLa cells do not express the Cx43 protein. Thus, the transfection of HeLa cells with Cx43 conjugated with enhanced green fluorescent protein (Cx43-EGFP) allows the analysis of GJ channel and hemichannel formation. By conventional fluorescence microscopy and TIRF microscopy, we determined the hyperthermia-induced changes in GJ plaque size and membranous hemichannel density, respectively. We also measured changes in LY transfer between several contiguous cells in a row, as described previously [57, 58], and changes in total Cx43-EGFP protein expression by WB.

**Figure 4:**
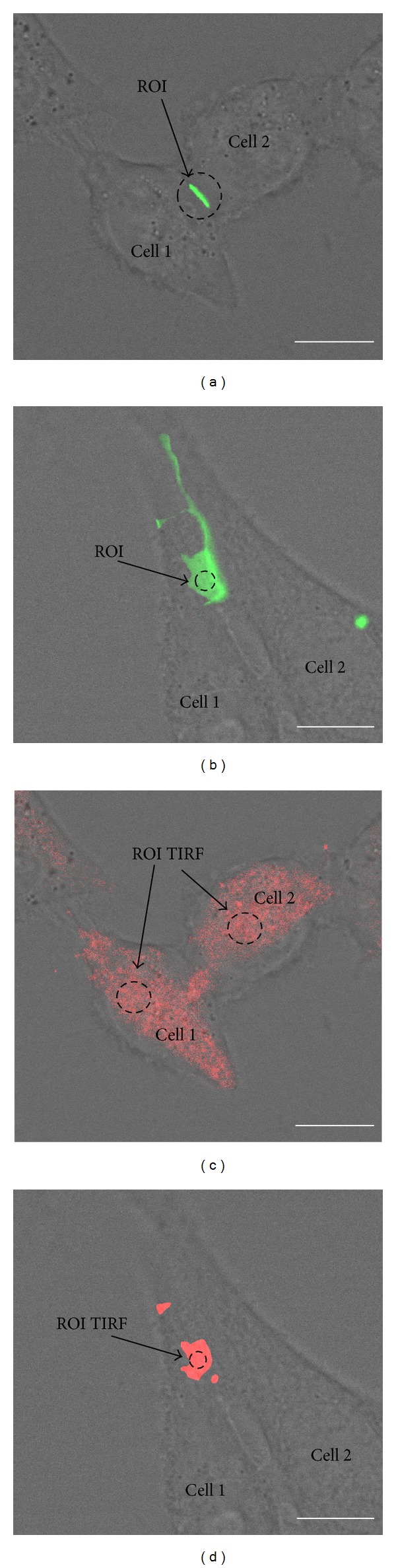
Measurement of Cx43-EGFP GJ channel number in JPs and hemichannel density in the plasma membrane of HeLa cells. (a) To estimate the total fluorescence of Cx43-EGFP JP (green), emitted light was integrated over the ROI encircling JP. (b) To estimate fluorescence intensity of the single Cx43-EGFP GJ channel, emitted light was integrated over the ROI situated in the horizontal JP of overlapping cells. (c) TIRF microscopy was used to estimate the density of Cx43-EGFP hemichannels (red) on the cell surface. Emitted light was integrated over the ROI situated in the site with most homogenous fluorescence. (d) To estimate the fluorescence intensity of the single Cx43-EGFP GJ hemichannel, emitted light was integrated by a TIRF microscope over the ROI situated in the horizontal JP of overlapping cells. Details are presented in the main text. Scale bar 10 *μ*m.

JPs varied in size from small puncta with a diameter of less than 1 *μ*m to very large JPs, up to ~10 *μ*m in length, occupying almost the entire region of the cell-cell contact (Figure 4(a)). To determine the number of GJ channels in HeLaCx43-EGFP cells, initially, we searched for overlapping cell pairs that showed JPs in contact regions and the JP could be seen* en face*, as it is illustrated in Figure 4(b). Imaging at different focus planes showed that some of the selected JPs were large, oriented parallel to the focal plane, and exhibited nearly homogeneous fluorescence intensity. To obtain fluorescence per unit area, emitted light was integrated over a given area of uniform fluorescence (region of interest, ROI). Fluorescence per unit area in* en face* JPs (*F*_JP_) was evaluated in arbitrary fluorescence units, a.u. (background fluorescence measured outside the JP was subtracted). In all experiments, we used the same objective, light intensity, and exposure time for fluorescence imaging. We estimated fluorescence produced by a single GJ channel (*F*_*γ*_) using *F*_JP_ and the density of channel packing. It was assumed that each Cx43-EGFP channel occupied 100 nm^2^ (corresponding to 10 nm center-to-center in a square array) as it was shown in electron microscopy studies of junctions in fixed tissues [59, 60] or isolated gap junctions imaged in aqueous media by atomic force microscopy [61]. In the present study, *F*_JP_ was equal to 503 ± 14 a.u. per 1 *μ*m^2^ on average (*n* = 14). From the ratio, 503 a.u./10 000 channels per 1 *μ*m^2^, *F*_*γ*_ = 0.05 a.u. was calculated. Furthermore, the total fluorescence intensity of JPs (*F*_*T*_) was measured independently on their spatial orientation. *F*_*T*_ was estimated by measuring the total fluorescence in the ROI enclosing JP as it is shown in Figure 4(a) (background subtracted). The number of physical channels (*N*_*T*_) present in each JP was determined from the following ratio: *N*_*T*_ = *F*_*T*_/*F*_*γ*_. We found that under control conditions (37°C), JPs of various size, ranging from 1 to 12 *μ*m, contained 120 935 ± 21 288 (*n* = 73) channels.

In order to determine the density of hemichannels in HeLaCx43-EGFP cell membranes by TIRF (Figure 4(c)), we analyzed JPs that could be seen* en face* at the maximal proximity to coverslip surface (Figure 4(d)). To obtain fluorescence per unit area, we used plaques where fluorescence in the region of interest (ROI TIRF) was homogeneous. We estimated fluorescence produced by single hemichannel (Fh_*γ*_ = *F*_JP_/2) using the same density of channel packing as above. We found that *F*_JP_ was equal to 239 ± 6 a.u. per 1 *μ*m^2^ on average (*n* = 21). From the ratio, 239 a.u./20 000 hemichannels per 1 *μ*m^2^, we found that Fh_*γ*_ = 0.012 a.u. Further, we measured the total fluorescence intensity of ROI TIRF (Fh_*T*_, background subtracted, Figure 4(c)). The density of hemichannels (Dh_*T*_) on HeLa cell surface was determined from the following ratio: Dh_*T*_ = Fh_*T*_/Fh_*γ*_. Under control conditions, the Cx43-EGFP hemichannel density on HeLa cell surface was 1514 ± 69 (*n* = 236) per 1 *μ*m^2^.

**Figure 5:**
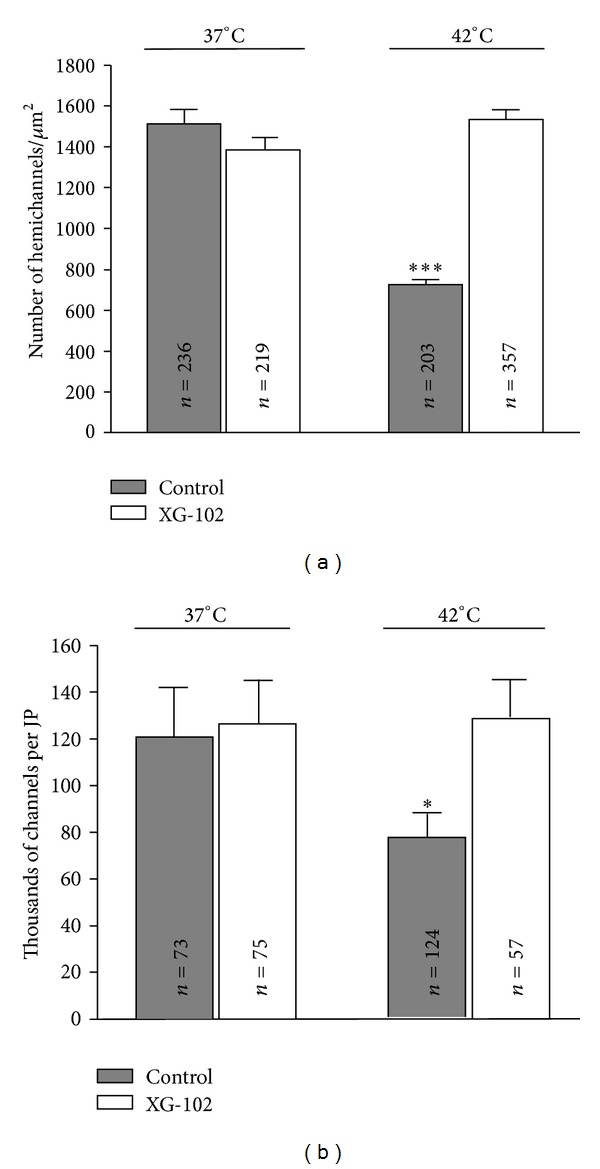
The effect of hyperthermia on the hemichannel density in Cx43-EGFP HeLa cell membranes (a) and number of channels in the JPs (b) in the absence and presence of JNK inhibitor XG-102 (4 *μ*M). *n* is indicated on the respective bar;  * = *P* < 0.05;  *** = *P* < 0.005, ANOVA followed by post hoc Bonferroni's test.

In the next set of experiments, HeLa cells were preincubated at 42°C for 2, 6, and 24 hours. Surprisingly, hyperthermia caused the opposite effects in HeLa cells compared with SkMs. The maximal effect of hyperthermia on Cx43-EGFP expression was achieved after 6 h; therefore, here we do not show respective results obtained after 2 and 24 hours of incubation under hyperthermic conditions. After 6 h of hyperthermic incubation, the number of Cx43-EGFP channels in JPs decreased by 34% (*P* < 0.05), and the density of hemichannels decreased by 52% (*P* < 0.005) (Figure 5).

Preincubation of HeLa cells at 37°C with XG-102 (4 *μ*M) had no effect on the number of Cx43-EGFP channels in JPs and on the density of hemichannels (Figure 5). However, the inhibition of JNK completely prevented the effect of hyperthermia; that is, the density of Cx43-EGFP hemichannels and the number of GJ channels were almost identical as in untreated controls at 37°C or 42°C.

Again, the inhibition of basal JNK activity at 37°C did not affect these parameters suggesting that hyperthermia activates different signal cascades as it does at the normal physiological temperature.

### 3.5. The Effect of Hyperthermia and JNK on Cx43 GJ Permeability in HeLa Cells

**Figure 6:**
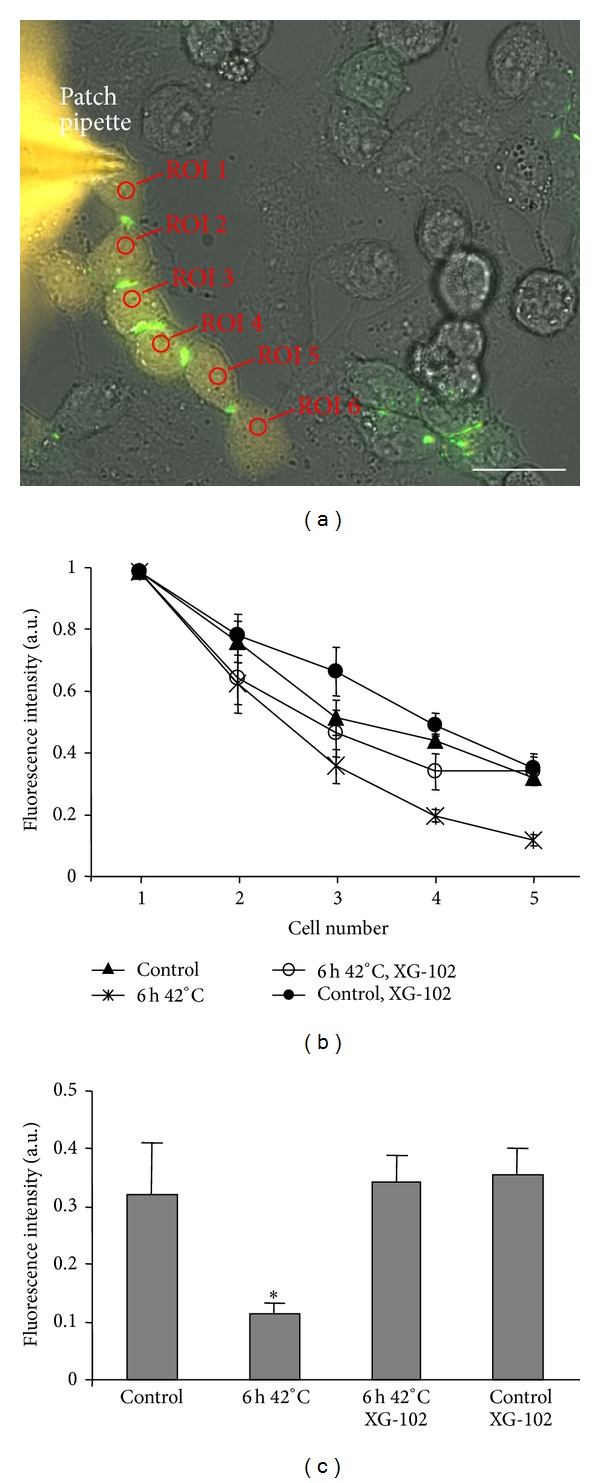
The effect of hyperthermia on Cx43-EGFP GJIC in HeLa cells. (a) LY was introduced through the patch pipette into the first cell of six cells in a row. (b) Kinetics of LY diffusion under different conditions. (c) LY fluorescence intensity in the fifth cell 3 min after its introduction into the first cell under control and hyperthermic conditions in the absence and presence of XG-102 (4 *μ*M). *n* = 8 in each set of experiments. Scale bar 25 *μ*m.  * = *P* < 0.05, ANOVA followed by post hoc Dunnet's test.

To evaluate the effect of hyperthermia on GJIC, LY was introduced through the patch-pipette into the first cell, and diffusion of LY was measured in at least 5 cells in a row with Cx43-EGFP GJ plaques between them (Figure 6(a)). The experiments were carried out under control (37°C) and hyperthermic conditions (6 h, 42°C) with and without XG-102 (4 *μ*M). Hyperthermia slowed down the LY diffusion between cells through GJs (Figures 6(b) and 6(c)). Under hyperthermic conditions, LY fluorescence in the fifth cell after 3 min from its introduction into the first cell was 2.9-fold lower compared with control (*P* < 0.05). XG-102, a JNK inhibitor, did not affect GJIC under control conditions but completely abolished the effect of hyperthermia on GJIC.

### 3.6. The Effect of Hyperthermia and JNK on Cx43 Protein Expression in HeLa Cells

**Figure 7:**
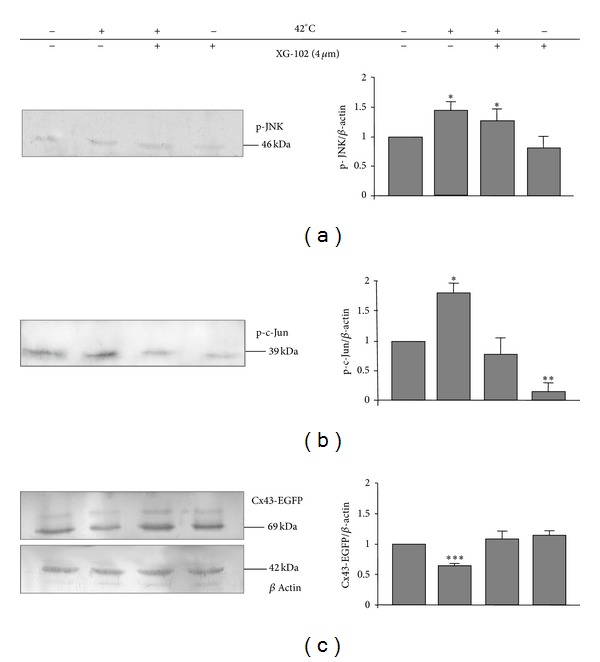
Effect of hyperthermia on the total expression of p-JNK (a), p-c-Jun (b), and Cx43-EGFP proteins (c) in HeLa cells. *n* = 3 in each set of experiments;  * = *P* < 0.05;  ** = *P* < 0.01;  *** = *P* < 0.005, Student's *t* test.

In HeLa cells, hyperthermia enhanced the activity of JNK and c-Jun by 1.4- and 1.8-fold, respectively (*P* < 0.05) (Figures 7(a) and 7(b)), similarly as seen in SkMs (Figures 3(a) and 3(b)). In contrast to SkMs, hyperthermia reduced the expression of Cx43-EGFP by ~35% (*P* < 0.005). XG-102 did not change the effect of hyperthermia on JNK phosphorylation but completely abolished the increase in phosphorylated c-Jun and a decline in Cx43-EGFP expression (Figures 7(b) and 7(c)). Under control conditions, XG-102 decreased the basal activity of c-Jun to ~20% of control (*P* < 0.01) but did not change the basal expression of Cx43.

Thus, in SkMs (Figure 3) and HeLa cells (Figure 7), hyperthermia induced JNK activation that exerted the opposite effects on Cx43 expression.

### 3.7. Impact of Hyperthermia on Actin Cytoskeleton in HeLa Cells and SkMs

Hemichannels from the Golgi apparatus are delivered to the plasma membrane through the tubulin and actin network, and this delivery can be affected by inflammation and heat shock [62, 63]. JNK could also be involved into remodeling and polymerization of F-actin underlying changes in cell motility [64]. Moreover, the changes in Cx expression and GJIC could affect the structure of the cytoskeleton [65]. Therefore, we wanted to know to which extent hyperthermia and JNK signaling are involved in actin cytoskeleton organization.

**Figure 8:**
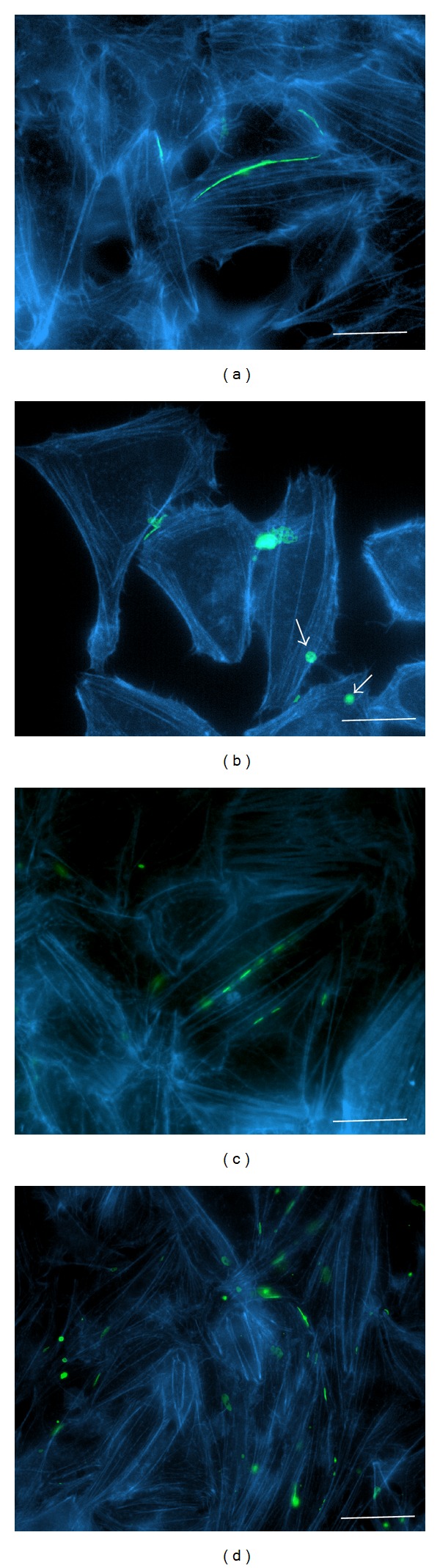
Impact of hyperthermia on actin filament organization in HeLa cells expressing Cx43-EGFP under control conditions (a); after hyperthermia (6 h at 42°C) (b); under control conditions with XG-102 (4 *μ*M) (c); after hyperthermia (6 h at 42°C) with XG-102 (4 *μ*M) (d). Blue: F-actin labeled with Alexa Fluor 594 phalloidin; green: Cx43-EGFP; white arrows indicate annular junctions containing internalized GJ plaques. Scale bar 25 *μ*m.

*HeLa Cells.* The exposure of HeLa cells expressing Cx43-EGFP to 42°C for 6 h drastically changed the actin filament network. Under control conditions, 4–6 thick integral actin filaments can be detected along HeLa cells (Figure 8(a)). After hyperthermia treatment, no actin filament in the center of the cells was observed, and only a few cells contained 1-2 actin filaments near the cell borders (Figure 8(b)). Since under hyperthermic conditions, the total fluorescence of actin did not change, we assume that the ratio of polymerized/nonpolymerized forms of actin was modified. The actin remodeling under hyperthermic conditions was substantially prevented by XG-102 (Figures 8(c) and 8(d)).

**Figure 9:**
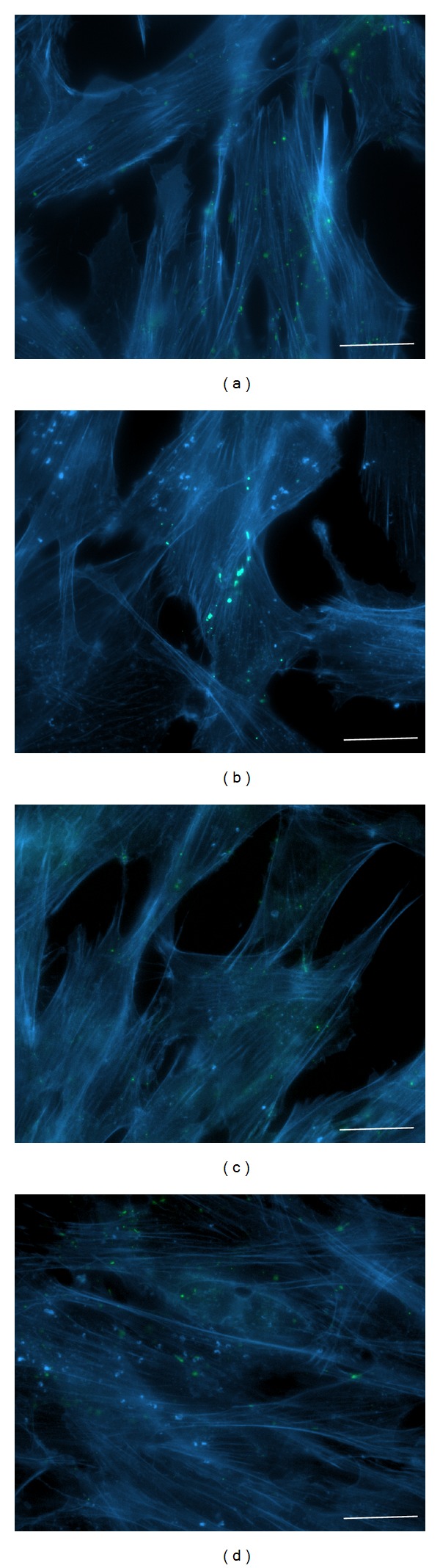
Impact of hyperthermia on actin filament organization in SkMs under control conditions (a); after hyperthermia (6 h at 42°C) (b); under control conditions with XG-102 (4 *μ*M) (c); after hyperthermia (6 h at 42°C) with XG-102 (4 *μ*M) (d). Blue: F-actin labeled with Alexa Fluor 594 phalloidin; green: Cx43-EGFP. Scale bar 25 *μ*m.

*SkM Cells.* In contrast, actin filaments remained intact and formed an integral actin network in SkMs after 6 h incubation at 42°C (Figure 9). XG-102 had no impact on the SkM actin network under control or hyperthermic conditions.

## 4. Discussion

The present study demonstrates that (i) in SkMs, hyperthermia induced the expression of Cx43 and enhanced the efficiency of GJIC but did not affect the actin cytoskeleton; (ii) in Cx43-EGFP-transfected HeLa cells, hyperthermia reduced the Cx43-EGFP expression and the efficiency of GJIC and disrupted the actin cytoskeleton. These opposite effects could be both antagonized by the inhibition of JNK/c-Jun stress pathway, which was activated by hyperthermia. These data support the idea that MAPK signaling may exert divergent, even opposite, effects depending on the cellular context.

### 4.1. JNK Mediate the “Positive” Effects of Hyperthermia in SkMs

In SkMs, hyperthermia increased the expression of Cx43 protein and improved GJIC, whereas the actin cytoskeleton was insensitive to hyperthermia. It is known that the stress-activated protein kinase JNK pathway can be activated upon the exposure of mammalian cells to hyperthermic stress [66]. For example, heat shock improves resistance to hypoxia-reoxygenation injury* in vitro* and enhances the survival of SkMs engrafted into the heart [33]. Impaired cell-to-cell coupling correlates with reduced Cx43 expression [67] and vice versa Cx43 upregulation improves GJIC [68]. However, the total Cx43 protein amount and even the total channel number in JPs are not sufficient indicators of the efficiency of cell-to-cell communication because only very small fraction of channels in JPs is functional for yet unknown reasons [69, 70]. Many other factors, such as transjunctional voltage, intracellular Ca^2+^, pH, and phosphorylation, also contribute to the gating and permeability properties of GJ channels [71–75]. The resistance of actin cytoskeleton to hyperthermia in SkMs could contribute to therapeutic effects of hyperthermia since actin filaments have been shown to be involved in the trafficking of Cx43 hemichannels to the plasma membrane [76]. In addition, the actin cytoskeleton may affect GJIC indirectly by targeting kinases, phosphatases, and other regulatory molecules to GJs [77].

### 4.2. JNK Mediate the “Negative” Effects of Hyperthermia in HeLa Cx43-EGFP Cells

In HeLa cells, hyperthermia diminished Cx43 protein levels, size of Cx43 GJ plaques, density of membranous Cx43 hemichannels, and efficiency of GJIC. These changes were blocked by the inhibition of JNK. JNK plays a major role in the cellular response to stress induced by inflammation and this is also true in myocardial I/R [11, 78, 79]. Controversial results were reported in different cell types concerning JNK activation, Cx43 expression, and Cx43 GJIC depending on whether pro- or antiapoptotic signaling pathways were activated [80]. While JNK mediated apoptosis in adult rat myocytes [81], it demonstrated antiapoptotic properties associated with the Akt signaling pathway in neonatal rat cardiomyocytes after I/R [41]. Interestingly, infarction size and lesions were smaller in the rat heart, when the JNK activation pathway was blocked by XG-102 [44]. The divergent effects of JNK actions can be mediated by individual isoforms and specific signalosomes activated in different cellular compartments. The improvement of Cx43-mediated intercellular communication possibly could have protective functions in the ischemic heart due to improved homeostasis. On the other hand, GJs may transmit cytotoxic materials as well [82], and consequently, the administration of GJ blockers might reduce the infarction size [83].

The role of JNK in the regulation of Cx43 expression in cardiac myocytes is also ambiguous. For instance, the inactivation of the JNK/c-Jun pathway decreased Cx43 expression in the cardiomyocytes of transgenic animals lacking MKK4 (JNK kinase) after phenylephrine stimulation [84], or JNK activation after amphetamine application increased the expression of Cx43 protein in cardiomyocytes [51]. In contrast, JNK activation mediated the downregulation of Cx43 expression in cardiomyocytes [52]. There are no data available on the JNK-dependent regulation of Cx43 protein expression in SkMs; however, the proliferation and differentiation processes in these cells may depend on the variations of Cx43 expression [85, 86].

### 4.3. JNK Inhibition Effectively Blocks the Outcomes of Hyperthermia

The inhibition of JNK by XG-102, a small-molecule inhibitor, was specific in two aspects. First, it did not affect the phosphorylation of JNK, since XG-102 blocks the binding domain but not the phosphorylated activation domain. Second, XG-102 inhibited the JNK-dependent activation, that is, the phosphorylation of c-Jun. XG-102 blocked hyperthermia-induced “positive” outcomes in SkMs and “negative” outcomes in Cx43-transfected HeLa cells. Thus, the signal-transcription coupling apparently differs between SkMs and Cx43-transfected HeLa cells. Also, the exogenous Cx43-EGFP might be differently more coupled to the JNK pathway than endogenous Cx43 proteins. However, in Cx43-EGFP-transfected SkMs, hyperthermia had a similar effect on GJIC as in wild type SkMs (our unpublished observation). These findings are in line with the concept of individual biological JNK signaling. Not only JNK isoforms (JNK1, 2, and 3) differ in their actions, but also one isoform can exert opposite actions depending on its intracellular localization and position within a given signalosome. In this context, the upstream MKK signaling with its spliced isoforms is an essential conductor for the orchestration of context-specific cellular responses [50, 87].

JNK has a pronounced basal activity, which contributes to normal cell functionality including proliferation [50, 88]. However, for the modulation of Cx43 expression and GJIC, much higher activity of JNK was required since the inhibition of basal activity of JNK by XG-102 had only a minor effect on these parameters whereas the hyperthermia-induced changes were blocked by XG-102. Also, in HeLa cells and SkMs, different JNK isoforms may realize JNK actions. We observed only one 46-kDa band in the lysates of HeLa cells, but typical 46- and 56-kDa (indicating p-JNK1 and p-JNK2) bands in the SkM samples.

### 4.4. Cytoskeleton and Hyperthermia

A close interaction between Cx43 and F-actin was demonstrated in various studies. For instance, on the one hand, cytochalasin B, a F-actin disrupter, was shown to inhibit Cx transport and GJ assembly [89] and vice versa GJ inhibitors (octanol, 18*β*-glycyrrhetinic acid) caused the discordance of actin stress fibers in embryonic rat astrocytes [90]. The functional blockade of GJ impaired F-actin fiber alignment and consequently reduced the migration of breast cancer cells [91]. Since hyperthermia may trigger the rearrangement of cytoskeleton proteins [92, 93], it could modify the Cx43 trafficking to the plasma membrane and GJ plaques. We demonstrate that the same hyperthermic conditions differently affected the F-actin network in HeLa cells and SkMs: after incubation of cells for 6 h at 42°C, the actin cytoskeletal network was disrupted in HeLa cells but remained intact in SkMs. These findings confirm previous observations that, depending on the cellular context and the type of stress stimuli [92, 93], the individual effects are modulated by different JNK isoforms [54, 94, 95].

Contradictory results can be found in the literature concerning the effect of inflammation and/or heat stress on Cx43 expression levels. For instance, after the exposure of neonatal rat cardiomyocytes to 43.5°C for 30 min, a drastic degradation of Cx43 was observed [96], while in human HE49 fibroblasts, the Cx43 expression level remained unchanged even after 8 h preincubation at 43°C [97]. Repeated heat shock does not alter Cx43 expression, possibly due to protective activity of Hsp70. Another heat shock protein Hsp72 was associated with increased resistance to hypoxia in rat L6 myoblasts [33]. The incubation of L6 cells at 42°C for 1 hour before transplantation into the heart increased the viability of preconditioned cells. An increase in Cx43 expression and intercellular communication under hyperthermic conditions might facilitate SkM integration into the damaged myocardium, presumably due to improved electrical communication between SkMs and cardiac myocytes. In turn, the reduction of Cx43 expression and impairment of GJ communication in cancer cells is closely related with the disruption of the actin filament network. A number of carcinogenesis studies have shown that levels of Cx43 or other connexin GJs are downregulated in cancer cells; decreased GJIC is considered as an important factor in oncogenesis [98–100] as well as the increased cytoplasmic pool of Cxs when Cx protein synthesis remains unchanged but connexon trafficking to the plasma membrane is inhibited due to disruption of the actin network [76]. In our experiments with HeLa cells, the decreased Cx43 GJ plaque size and hemichannel density after hyperthermic treatment clearly correlated with the breakdown of the actin network and possibly with a decrease in protein synthesis.

For many years hyperthermia has been known as a therapeutic tool for the treatment of certain cancer types [101], because cancer cells are particularly sensitive to hyperthermia [102]. Our finding that the effects of moderate hyperthermia on the cytoskeletal structure differ in normal SkMs and cancer (HeLa) cells indicate that molecular mechanisms underlying the sensitivity of cellular response to hyperthermia are complex and involve cell type-dependent changes of membrane properties, signaling pathways, ion homeostasis, protein expression, ROS generation, redox disbalance, and possible energy crisis due to mitochondrial dysfunction [103–105].

## 5. Conclusions

- In SkMs, hyperthermia had no effect on the actin filament network and increased the total amount of Cx43 and the efficiency of GJIC. Thus, hyperthermia pretreatment of SkMs may improve their efficiency in the strategies of heart regeneration.
- In Cx43-EGFP-expressing HeLa cells, hyperthermia decreased the total amount of the Cx43-EGFP protein, number of channels in GJ plaques, density of hemichannels in the cell membranes, and efficiency of GJIC. These effects correlated with the downregulation of the actin filament network.
- JNK is a central mediator of hyperthermia-induced cellular responses in both SkMs and HeLa cells.
- The comparison of SkMs and HeLa cells provides an attractive model to identify the regulatory players within signalosomes, which determine the cell-specific outcomes of hyperthermia.

## Abbreviations

Cx43: Connexin 43
Dh_*T*_: Density of physical hemichannels
EGFP: Enhanced green fluorescent protein
ERK1/2: Extracellular-signal-regulated kinases 1/2
FD_50_: Exponential decay constant estimated as a time after which dye fluorescence was equal to half of maximal
*F*_JP_: Fluorescence per unit area in* en face* JPs
*F*_*γ*_: Fluorescence produced by single GJ channel
Fh_*γ*_: Fluorescence produced by single hemichannel
Fh_*T*_: Total fluorescence intensity of ROI TIRF
GJ: Gap junction
GJIC: Gap junction intercellular communication
I/R: Ischemia/reperfusion
JNK: c-Jun NH_2_-terminal kinase
JP: Junctional plaque
LY: Lucifer yellow CH dilithium salt
MAPK: Mitogen activated protein kinase
MI: Myocardial infarction
*N*_*T*_: Number of physical channels
ROI: Region of interest
p-c-Jun: Phosphorylated c-jun
p-JNK: Phosphorylated JNK
SkM: Skeletal myoblast
TIRF: Total internal reflection fluorescence
VEGF: Vascular endothelial growth factor
WB: Western blot.
